# Applicability of metabolomics to improve sustainable grapevine production

**DOI:** 10.3389/fmolb.2024.1395677

**Published:** 2024-09-06

**Authors:** Catarina Estêvão, Lénia Rodrigues, Ana Elisa Rato, Raquel Garcia, Hélia Cardoso, Catarina Campos

**Affiliations:** ^1^ MED—Mediterranean Institute for Agriculture, Environment and Development & CHANGE—Global Change and Sustainability Institute, Institute for Advanced Studies and Research, Universidade de Évora, Pólo da Mitra, Évora, Portugal; ^2^ MED—Mediterranean Institute for Agriculture, Environment and Development & CHANGE—Global Change and Sustainability Institute, Departamento de Fitotecnia, Escola de Ciências e Tecnologia, Universidade de Évora, Pólo da Mitra, Évora, Portugal; ^3^ MED—Mediterranean Institute for Agriculture, Environment and Development & CHANGE—Global Change and Sustainability Institute, Departamento de Biologia, Escola de Ciências e Tecnologia, Universidade de Évora, Pólo da Mitra, Évora, Portugal

**Keywords:** plant metabolome, *Vitis vinifera* L., analytical tools, stress tolerance, acclimatization, plant plasticity

## Abstract

Metabolites represent the end product of gene expression, protein interaction and other regulatory mechanisms. The metabolome reflects a biological system’s response to genetic and environmental changes, providing a more accurate description of plants’ phenotype than the transcriptome or the proteome. Grapevine (*Vitis vinifera* L.), established for the production of wine grapes, table grapes, and raisins, holds immense agronomical and economic significance not only in the Mediterranean region but worldwide. As all plants, grapevines face the adverse impact of biotic and abiotic stresses that negatively affect multiple stages of grape and wine industry, including plant and berry development pre- and post-harvest, fresh grapes processing and consequently wine quality. In the present review we highlight the applicability of metabolome analysis in the understanding of the mechanisms involved in grapevine response and acclimatization upon the main biotic and abiotic constrains. The metabolome of induced morphogenic processes such as adventitious rooting and somatic embryogenesis is also explored, as it adds knowledge on the physiological and molecular phenomena occurring in the explants used, and on the successfully propagation of grapevines with desired traits. Finally, the microbiome-induced metabolites in grapevine are discussed in view of beneficial applications derived from the plant symbioses.

## 1 Metabolomics unrevealing plant phenotypes—agronomical traits

In both natural and cultivated ecosystems, plants, as sessile organisms, are exposed to a wide range of environmental stresses throughout their lifespan. Adverse external conditions can significantly impact plant growth, development, and consequently, reduce the productivity of agricultural crops (Gull et al., 2019). Different genotypes present different abilities to efficiently express the different phenotypes, which are determined by differences at genomic level or regulated by epigenetic events. Grapevine (*Vitis vinifera* L.), established for the production of wine grapes, table grapes, and raisins, holds immense agronomical and economic significance not only in the Mediterranean region but worldwide. Grapevines growing in vineyards face the adverse impact of biotic and abiotic stresses that negatively affect multiple stages of grape and wine industry, including plant and berry development pre- and post-harvest, fresh grapes processing and exportation, and also wine quality.

**FIGURE 1:**
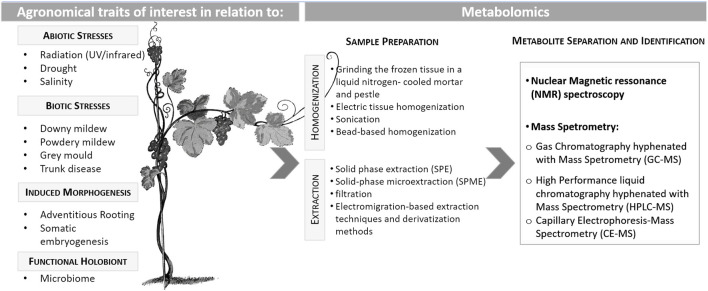
Different grapevine agronomical traits that have been investigated through metabolome analysis and the methods considered for those analyses.

Environmental stresses are usually categorized as biotic stress if caused by any living organism (e.g., viruses, bacteria, fungi, oomycetes, nematodes, insects, arachnids, etc.) responsible for a plant disease or pest attack. Those biotic stress agents are a prominent factor responsible for grapevine losses, as they can directly and indirectly deprive host plants of nutrients which can ultimately result in plant death (Gull et al., 2019; Li et al., 2019). On the other hand, abiotic stresses, such as temperature (cold or freezing/heat), water availability (drought/flooding), high salinity, heavy metals, and ultraviolet radiation, are considered the environmental abiotic factors with a great negative impact on crop yield worldwide. Plants can develop different biochemical and physiological strategies to counteract and mitigate the detrimental effects of those biotic and abiotic stresses. Plant responses against environmental stresses are determined by intricate regulatory mechanisms that involve differential gene expression, post-transcriptional regulation, protein synthesis and modification and the accumulation of metabolites (Gull et al., 2019; Li et al., 2019). The development of multistress-tolerant genotypes is nowadays the main task of grapevine breeders, aiming to contribute to more sustainable agricultural production (Figure 1). One of the breeding strategies is based on controlled crosses involving tolerant/resistant progenitors with high plasticity, a trait recognized as the efficient capacity to acclimatize to undesired conditions (Cardoso et al., 2013).

Metabolites represent the end product of gene expression, protein interaction and other regulatory mechanisms. The metabolome reflects a biological system’s response to genetic and environmental changes, providing a more accurate description of a plant phenotype than the transcriptome or the proteome (Arbona et al., 2013; Fiehn, 2002; Pinu, 2018). Research on plant metabolome provides important knowledge on the metabolic processes of plants, playing an important role in enhancing plant resistance to stresses and improving crop quality and yield (Li et al., 2019). Metabolomic approaches are designed to identify and quantify all metabolites in a biological system (organism, tissue, cell, or cell compartment) at a given period of time (Li et al., 2019; Arbona et al., 2013; Fiehn, 2002). Through targeted metabolomic analyses of host-pathogen interactions, several mechanisms of plant resistance to biotic stresses have been unveiled. Comprehensive or untargeted analyses allow the detection of a wide range of compounds that can be related to plant resistance to biotic stress, which can then be mapped in their metabolic pathways to provide a better understanding of their regulation and the identification of the corresponding genes (Kushalappa and Gunnaiah, 2013).

Induced plant morphogenesis is an agronomical trait that involves a cell reprogramming process ending with *de novo* differentiation of a new organ. *De novo* morphogenesis is described as a response to abiotic stresses (Cardoso et al., 2013; Arnholdt-Schmitt et al., 2016) and includes processes with high agronomical impact, such as the development of adventitious roots in recalcitrant species/cultivars, and the differentiation of somatic embryos for the propagation of selected clonal lines. The improvement of these processes offers therefore important advantages for grapevine breeding. Metabolomic studies of induced morphogenic processes are already taking place for several plant species and are expected to provide a theoretical basis for improving rooting and propagation rates of interesting cultivars.

The microbial communities of plants, also known as the plant microbiome, are found in the soil surrounding the roots, the above ground surface and endosphere (Trivedi et al., 2020). Plant microbiomes contain both beneficial and pathogenic microbes, and the characterization of plant microbiomes and their relation with the host plants using metabolomics is becoming a hot topic in research, as these interactions can greatly impact plant phenotypic responses. For example, microbes adapted to different locations can induce the same plants to produce different secondary metabolites (Köberl et al., 2013). The understanding of the plant-microbe interactions can therefore be directed for instance to i) enhance the production of grapevine desirable metabolites or ii) select plants with specific metabolites that can attract beneficial microbes.

## 2 Introduction to analytical techniques in plant metabolomic analysis

Metabolomics is a multidisciplinary field comprising a comprehensive study of small-molecules belonging to biological systems, like cell, tissue, organ, biological fluid, or organism, enabling the identification and semi-quantification of all known and unknown metabolites–usually small molecules with a weight below 1,500 Da. A huge coverage of the metabolome is very challenging owing to the broad range of physicochemical properties of those molecules. Regarding compounds identification, metabolomics research relies on confidence levels, having been defined from identified metabolites (Level 1), putatively annotated compounds (Level 2), putatively characterized compound classes (Level 3) and unknown (Level 4) (Reisdorph et al., 2020). Commonly, the metabolomic study includes a sample preparation step, followed by the identification and quantification of metabolites using spectroscopic (NMR) and spectrometric (MS) techniques, and separation techniques coupled to MS detection, such as chromatographic (LC, GC) techniques, and more recently, comprehensive analytical techniques (Reisdorph et al., 2020). Depending on the objective of the study, a common experimental approach includes targeted and untargeted metabolomics strategies. Targeted approaches focus on the analysis of a predefined set of metabolites. On the other hand, an untargeted metabolite analysis aims to detect as many distinct metabolites as possible, without a prior knowledge of their characteristics (Patel et al., 2021).

### 2.1 Sample preparation–a key step

Generally, metabolomic research is mostly conducted in complex matrices, therefore a previous step of sample preparation is required to isolate and pre-concentrate the compounds under study. It is also crucial to remove compounds that could cause interference in the analysis process. Particularly, the use of combined sample preparation techniques enables, in a single step, to achieve satisfactory extraction efficiency, cleanup, as well as excellent preconcentration capacity, providing a pathway to tackle complex matrix applications, such as metabolomics.

Sample preparation is a key step in metabolomic study, starting with the careful collection of samples to prevent metabolic alterations. Immediately after collection, it is essential to stop all metabolic activity, using liquid nitrogen for this purpose. After that, samples should be stored at low temperatures, normally at −80°C, to preserve their metabolic state until further analysis. In order to make extraction more efficient, powdering is another important step, contributing to the increase of surface area. Metabolites can be isolated from the biological matrix using various extraction methods. Solvent extraction is a widely used technique, and the choice of solvents is also crucial, as a single solvent cannot extract the entire variety of metabolites. To solve this, a broad spectrum of metabolites, including polar, nonpolar and hydrophilic compounds can be isolated using a solvent system composed of chloroform: methanol: water (Patel et al., 2021). In most cases, after extraction, the sample is concentrated by vacuum-drying.

In fact, sample preparation techniques could provide three different achievements: (i) sample/analyte transformation into a form suitable for measurement; (ii) simplifying the matrix; and (iii) preconcentration to improve detection limit. Indeed, the sample preparation step is considered mandatory, even when using advanced instrumental techniques, since the analyte’s accurate determination at the trace level without any pretreatment step is very challenging (Kanu, 2021).

### 2.2 Separation and identification techniques

#### 2.2.1 Nuclear magnetic resonance spectroscopy

Based on the specific nuclear spin of atom nuclei when exposed to an external magnetic field, Nuclear Magnetic ressonance (NMR) spectroscopy can quantitate and identify a wide range of compounds from metabolome. This technique is largely used on metabolomic applications due to its high reproducibility and cost efficiency. Nevertheless, it requires that the compounds have a concentration at micromolar range, which is higher than the required for mass spectrometry (MS) techniques. Due to its simple sample preparation and brief analysis time, this technique is suitable for untargeted metabolomics aproach. Therefore, it is also used as complementary technique to GC-MS and LC-MS. However, the major limitation of NMR is its significantly lower sensitivity compared to MS (Markley et al., 2017), making it inappropriate for the analysis of large numbers of low-abundance metabolites.

#### 2.2.2 Mass spectrometry (MS)

Mass spectrometry is a valuable tool in high-throughput metabolomics, being widely used on this application due to its high sensitivity and wide range of covered metabolites. MS has a higher resolution and can be coupled with chromatographic techniques, becoming an even more powerful analytical tool. Indeed, the developments and improvements in mass sensitivity have expanded the range of metabolites that can be analysed by MS leading to an increase in the accuracy of compound identification (Kanu, 2021; Zhang et al., 2016).

High-resolution mass spectrometers, such as Fourier transform (FT) mass spectrometers, which include FT ion cyclotron resonance (FT-ICR), started to be used in untargeted metabolomics approaches since 2002, to identify metabolic changes during the development of strawberry fruit and in tobacco flowers (Aharoni et al., 2002), triggering a new era for plant metabolomics. This technique has a high capability to analyse complex samples, detecting and discriminate hundreds to thousands of metabolites, and can be used through direct sample injection, without chromatographic separation, or coupled with various chromatographic techniques. Several FT-ICR-MS–based plant studies have emerged involving different plant species, such as *Arabidopsis thaliana, V. vinifera*, *Allium cepa, Medicago truncatula, Solanum lycopersicum* and *Nicotiana tabacum* (Maia et al., 2023).

#### 2.2.3 Gas chromatography hyphenated with mass spectrometry (GC-MS)

Another powerful instrumental analytical technique for metabolomic studies is GC-MS since its provides a platform for non-targeted efficient and reproducible analysis. An requirement of this technique is the volatility of the sample. An advanced GC system equipped with time-of-flight mass spectrometry (TOF-MS) analyser proves to be promising for metabolic profiling reaching higher mass accuracy, faster acquisition times, and improved deconvolution for complex mixtures (Patel et al., 2021).

#### 2.2.4 High performance liquid chromatography hyphenated with mass spectrometry (HPLC-MS)

In metabolomic field, a liquid chromatographic-based hyphenated technique—HPLC-MS—has proven its usefulness for metabolite characterization and structural information. This analytical tool is suitable to characterize unknown endogenous or exogenous metabolites present in complex biological samples. Due to the high sensitivity, robustness, and quantitative reproducibility, LC-MS represents a remarkable tool for metabolomic applications.

#### 2.2.5 Capillary electrophoresis-mass spectrometry (CE-MS)

The separation of ionic metabolites based on their charge and size ratio is performed by CE-MS, displaying several advantages, namely: the requirement of small injection volumes, a fast and high resolution of charged compounds and an accurate assessment of metabolites characterization based on mass fragmentation. Altogether, CE-MS has proven to be a relevant platform for plant metabolome studies (Patel et al., 2021).

In summary, a huge improvement on the field of plant metabolomics has ocurred provided by the use of conventional techniques, like HPLC, GC, NMR and moreover the development of multiplatforms containing complementary tecniques, enabling both untargeted and targeted analysis. Generally, those techniques are able to assess the levels of endogenous metabolites, which allows monitoring and staging of several diseases as well as systemic responses to environmental, therapeutic, or genetic interventions.

## 3 Metabolomics application in grapevine stress studies

### 3.1 Biotic stress

Diseases caused by fungi, oomycetes, and viruses pose the greatest threats to overall plant development, yield, and fruit quality. The susceptibility of most grapevine cultivars to biotic agents increases the risk of substantial economic losses (Armijo et al., 2016). In terms of severity, in Europe, the most concerning pests and diseases are Downy Mildew, Powdery Mildew, Grey Mold, Grape Trunk Diseases, and Virus-induced diseases such as GFLV fanleaf and GRLaV leafroll-associated viruses (Bois et al., 2017) (Table 1).

**TABLE 1:** Changes in grapevine metabolism in response to major biotic and abiotic stresses.

Biotic stress				
Stress	Metabolite	Change	Plant Material	References
Downy mildew disease	Lipids: AA, EPA, triacylglycerols	Increase in both susceptible and resistant cultivars	Leaves	Negrel et al. (2018)
	Inositol, glutamate, alanine, and caffeic acid	Increase in resistant cultivar	Leaves	Figueiredo et al. (2008)
	Quercetin-3-O-glucoside and a trans-feruloyl derivative	Increase in resistant cultivar	Leaves	Ali et al. (2009)
	Phenylpropanoids and flavonoids	Increase in resistant cultivar	Leaves	Ali et al. (2012)
	Caffeic acid 3-glucoside, oleic acid and palmitoleic acid	Increase in resistant cultivar	Leaves	Figueiredo et al. (2008); Maia et al. (2018); Lim et al. (2017)
Powdery mildew disease	Flavonoids	Increase in susceptible infected cultivar	Grapes	Yu et al. (2022)
	Stilbenes	Increase in resistant infected cultivar	Grapes	Yu et al. (2022)
	Phenylpropanoids and fatty acids	Increase in infected susceptible cultivar	Grapes	Pimentel et al. (2021)
	Gallic, eicosanoic, and docosanoic acids and resveratrol	Increase in infected cultivar	Grapes	Pimentel et al. (2021)
	Total phenolics content	Increase in both susceptible and resistant cultivars after infection	Leaves	Taware et al. (2010)
	Hydroxybenzoic acids, hydroxycinnamic acids, (±)-catechin derivatives, and stilbenes	Increase in both susceptible and resistant cultivars after infection	Leaves and grapes	Taware et al. (2010)
	Pallidol, oleic acid + cis vaccenic acid and astringin	Increase after infection	Leaves	Ciubotaru et al. (2023)
Grey mold disease	Gallic and azelaic acids, arabitol, ribitol, 4-amino butanoic acid, 1-O-methyl- glucopyranoside	Increase after infection	Grapes	Agudelo-Romero et al. (2015)
	Glycerol and fatty acids (such as 9-octadecenoic acid (Z), or oleic acid)	Increase after infection	Grapes	Agudelo-Romero et al. (2015)
	Sesquiterpenes	Increase after infection	Grapes	Schueuermann et al. (2019)
	Oligosaccharides	Increase after infection	Grapes	Hong et al. (2012)
	Glycerol, 2,3-butanediol, succinate and phenylpropanoids	Decrease after infection	Grapes	Hong et al. (2012)
Grapevine trunk diseases	Total phenolic content	Increase after infection	Leaves	Lima et al. (2017); Rusjan et al. (2017)
	Trans-caffeoyltartaric acid, trans-coumaroyl-tartaric acid, quercetin-3-O-glucoside, quercetin-3-O-galactoside, kaempferol-3-glucoside and myricetin	Increase after infection	Leaves	Lima et al. (2017)
	Stilbene polyphenols	Increase after infection	Leaves	Lima et al. (2017); Lemaitre-Guillier et al. (2020)
	Sugars (fructose, glucose, sucrose)	Decrease after infection	Woody tissue	Labois et al. (2020)
	Amino acids (aspartic acid, serine, and asparagine)	Decrease after infection	Woody tissue	Labois et al. (2020)
	Sugar alcohol content (mannitol and arabitol)	Increase after infection	Woody tissue	Labois et al. (2020)

Abiotic stress				
Stress	Metabolite	Change	Plant Material	References
Drought	Reactive oxygen species (ROS)	Increase	Leaves, roots, grapes	Vidigal et al. (2013)
	Antioxidant enzymes	Increase	Leaves, roots, grapes	Ju et al. (2018)
	Phenolic compounds	Increase	Grapes	Ollé et al. (2011); Pinasseau et al. (2017)
	Sacarose	Increase	Grapes	Grimplet et al. (2009)
	Proline	Increase	Leaves and grapes	Doupis et al. (2011); Hochberg et al. (2013); Ju et al. (2018); Deluc et al. (2009)
	Myo-inositol	Increase	Grapes	Grimplet et al. (2009)
	Green leaf volatiles	Increase	Leaves	Ju et al. (2018)
Salinity	Sugars	Decrease	Leaves	Lu et al. (2022)
	Organic acids	Increase	Leaves	Lu et al. (2022)
	Carotenoids and chlorophylls	Decrease	Leaves	Lu et al. (2022)
	Rubisco	Decrease	Leaves	Lu et al. (2022)
	Antioxidant enzymes	Increase	Leaves	Zhou-Tsang et al. (2021); Lu et al. (2022)
	Total phenolic content	Increase	Leaves, roots	Zhou-Tsang et al. (2021)
Ultra violet and infrared radiation	Flavonoid content	Increase	Grapes	Agudelo-Romero et al. (2015); Liu et al., (2015); Loyola et al. (2016); (Rouxinol et al. (2023)
	Anthocyanin	Decrease	Grapes	Matus et al. (2009)
	Sugar content	Decrease	Grapes	Matus et al. (2009)
	Sesquiterpene and monoterpenes	Increase	Grapes	Gil et al. (2012)
	Zeaxanthin, violaxanthin and anteraxanthin	Increase	Leaves	Gambetta et al. (2021)
	Stilbenes	Increase	Leaves	Kiselev et al. (2019)

#### 3.1.1 Downy mildew disease

Downy mildew is a highly destructive disease that poses a significant threat to viticulture, particularly in temperate-humid climates. It is caused by the biotrophic oomycete *Plasmopara viticola*, capable of damaging grapevine green tissues such as leaves, tendrils, bunches, and shoots (Buonassisi et al., 2017). Originally endemic to wild *Vitis* species in North America, *P. viticola* was first observed in Europe in 1878, most likely introduced through American grape cuttings used for replanting vineyards devastated by phylloxera. This accidental introduction into Europe had significant repercussions, resulting in extensive damage to the grape industry since, unlike American *Vitis* species, European *V. vinifera* cultivars are highly susceptible to *P. viticola* (Gessler et al., 2011). Without treatment, and under favourable weather conditions, this disease can cause devastating crop losses of up to 75% in a single season (Buonassisi et al., 2017). The use of modern fungicides in alignment with weather-based warning systems can effectively prevent the occurrence of damage by downy mildew (Gessler et al., 2011). However, the extensive and repeated use of fungicides raises environmental and health concerns in terms of pollution, development of resistant pathogen strains, residual toxicity, and pathogen pressure (Buonassisi et al., 2017). Despite this, alternatives to chemical treatments still have a limited role in the control of grapevine downy mildew disease. One example is the development of new resistant cultivars by following a conventional breeding approach using resistant progenitors (Buonassisi et al., 2017; Gessler et al., 2011).

Over the last few decades, many studies have utilized metabolomics approaches to gain insight into grapevine’s intricate defence mechanisms against *P. viticola*. Methods of metabolomic profiling have been used to uncover possible metabolite markers for downy mildew resistance and susceptibility traits (Batovska et al., 2009).

Research on the host-pathogen interaction between grapevine and *P. viticola*, by comparing healthy and inoculated samples, has supplied potential metabolite markers not only for phenotyping downy mildew resistance but also for disease diagnosis, identifying infected individuals and evaluating the disease progression. Major changes in metabolites are shown in Table 1. Becker and colleagues (Becker et al., 2013) were able to distinguish inoculated and healthy samples based on the statistical analysis of 19 compounds, however, they were unable to differentiate the samples based on their resistance to *P. viticola*, suggesting that these markers are likely involved in the defence response against the disease but unrelated to resistance traits. The changes in primary and secondary metabolism of resistant cultivar ‘Bianca’ were further investigated at different timepoints after inoculation with *P. viticola*, and 53 metabolites modulated in response to pathogen infection were identified and possibly used as biomarkers for different stages of the plants’ defence response (Chitarrini et al., 2017). Billet and co-workers (Billet et al., 2020) used a semi-targeted metabolomics approach to screen downy mildew symptomatic leaves in field conditions that allow them to identify biomarkers for specific disease symptoms. Furthermore, a non-targeted metabolomic approach characterized the compounds belonging to three families of atypical lipids, which are abundant in *P. viticola* sporangia and undetected in healthy grapevine leaf tissues (Negrel et al., 2018). The lipid analysis conducted at various time points after inoculation showed that the pattern of lipid accumulation was modified along the infection process, suggesting that the lipid profile may be used as an indicator of disease progression. Grapevine cultivars with different susceptibility showcased different patterns and timings of *Plasmopara*-specific lipids accumulation, indicating that they can also be used as markers for resistance. Furthermore, these lipids were easily detected in infected tissues at very early stages of the infection process (before sporulation), showing their potential to be used as markers for early detection of downy mildew infection, even before the appearance of visible symptoms (Negrel et al., 2018).

In a study combining transcript and metabolic profiling of grapevine leaves, the fungus-resistant cultivar ‘Regent’ was compared with the susceptible ‘Trincadeira’ (Figueiredo et al., 2008). The metabolic profile of both cultivars were analysed by NMR spectroscopy and multivariate data analysis. Results showed that the resistant cultivar accumulated compounds with known roles in pathogen defence or resistance, such as inositol, glutamate, alanine, and caffeic acid, that may confer ‘Regent’ a faster response against pathogen attack, providing its higher resistance.

Using similar NMR methodology, Ali and co-workers (Ali et al., 2009) managed to identify the major metabolites contributing to the discrimination between six different grapevine cultivars with various levels of resistance to downy mildew. The quantitative analysis of the metabolites responsible for discriminating the cultivars in terms of their resistance to downy mildew revealed that resistant cultivars exhibited higher levels of quercetin-3-O-glucoside and a trans-feruloyl derivative, suggesting their potential involvement in defence mechanisms. Further studies by Ali (Ali et al., 2012) compared the responses of the cultivars ‘Regent’ and ‘Trincadeira’ upon inoculation with *P. viticola*. The results indicated that infected plants adjust their metabolism, channelling resources towards the production of phenylpropanoids and flavonoids, and accumulating primary metabolites associated to stress response. The resistant cultivar ‘Regent,’ in contrast to susceptible ‘Trincadeira’ displayed a higher synthesis of phenolics and other stress-signalling related metabolites since the initial stages of infection. This accumulation of metabolites may represent the first response of ‘Regent’ against infection, suggesting their potential role as key elements in grapevine resistance to *P. viticola*.

More recently, it was possible to discriminate amongst the susceptible cultivar ‘Trincadeira’ and the resistant ‘Regent’ using an untargeted approach (FT-ICR-MS) (Maia et al., 2018). Among the discriminatory compounds in ‘Regent’ were caffeic acid 3-glucoside, corroborating previous findings (Figueiredo et al., 2008), oleic acid (18:1) and its methyl ester form, known for inducing the activation of defence responses, and palmitoleic acid (16:1), a fatty acid associated with lipid signalling (Lim et al., 2017). This method of metabolic profiling allowed a larger coverage of the metabolome and the observation of a higher number of cultivar-specific features when compared to the NMR analysis previously done (Figueiredo et al., 2008).

#### 3.1.2 Powdery mildew disease

Grapevine powdery mildew is one of the most threatening fungal diseases worldwide, especially in dry and warm climates. It is caused by the biotrophic ascomycete *Erysiphe necator* [syn. *Uncinula necator* (Schw.) Burr.] that has *V. vinifera* as its most economically important host (Armijo et al., 2016; Gadoury et al., 2012). This pathogen can infect all green tissues of its host, causing significant production losses, reducing yield and fruit quality by impacting berry sugar content and acidity (Armijo et al., 2016; Ferreira et al., 2004). Originating from North America, *E. necator* was introduced in Europe in the 1840s, causing substantial damage on European *V. vinifera* that is highly susceptible to infection by this pathogen (Armijo et al., 2016; Gadoury et al., 2012; Ferreira et al., 2004). Powdery mildew disease management strategies usually rely on chemical treatments, with the repeated use of fungicides, which has led to the selection of resistant pathogen populations, reducing the effectiveness in disease control.

Metabolomics studies are nowadays a valuable tool for designing new strategies and solutions to prevent and control powdery mildew disease, based on differences in tolerance (Yu et al., 2022). Yu and colleagues (Yu et al., 2022) used a metabolomic profiling approach to assess metabolome differences in berries of two grapevine cultivars: ‘Guipu’ No.6 (GP6), a *Vitis* sp., showing better powdery mildew leaf tolerance than ‘Marselan,’ a *V. vinifera* cultivar. Comparing healthy and infected samples, the authors revealed that there was an accumulation of flavonoids, phenolic acids, stilbenes, and terpenoids after infection, indicating that the defence mechanisms against powdery mildew of the two cultivars may be associated with phenylpropane-flavonoid metabolism. ‘GP6’ showed a higher accumulation of stilbenes, while ‘Marselan’ showed a higher accumulation of flavonoids, which suggests that the two cultivars employed different responses upon powdery mildew infection (Yu et al., 2022) (Table 1). In a research combining the transcriptome and metabolome analyses of grapes of *V. vinifera* cv. ‘Carignan’, highly susceptible to *E. necator* infection, healthy and naturally infected samples were compared (Pimentel et al., 2021). Defense mechanisms involving phytohormones like salicylic acid (SA) and jasmonates (JA) were activated, with the concomitant accumulation of metabolites such as phenylpropanoids and fatty acids. Even though the metabolic reprograming undertaken by this cultivar was not enough to restrict *E. necator* infection, several metabolites were significantly increased or detected only in infected berries, indicating their potential use as metabolic biomarkers of infection at early stages, under field conditions. Among these metabolites are gallic, eicosanoic, and docosanoic acids and resveratrol (Pimentel et al., 2021) (Table 1).

The content of total phenolics and individual phenolic groups in healthy and powdery mildew infected leaves, berries and wines of three different cultivars were also analysed (Taware et al., 2010). Positive relationship between the phenolic content of leaves and berries, and disease incidence and severity was observed. The total phenolics content of leaves increased with the powdery mildew infection in all the cultivars, with higher accumulation in the less susceptible cultivar. The changes in berry phenolics were reflected on corresponding wines, impacting their quality. This study revealed that, after infection, there was an accumulation of hydroxybenzoic acids (gallic acid, synergic acid and vanillic acid), hydroxycinnamic acids (caffeic acid and p-coumaric acid), (±)-catechin derivatives, and stilbenes (piceatannol and resveratrol) in leaves and berries, indicating their role in the defence against infection (Taware et al., 2010) (Table 1).

Using an untargeted approach, Maia and co-workers (Maia et al., 2020) compared different genotypes presenting different levels of resistance to downy and powdery mildews and black rot, without being submitted to any stress. This study revealed an enrichment in the flavonoid biosynthesis pathway. Of the metabolites responsible for the discrimination between resistant/partial resistant and susceptible genotypes, some of the identified compounds were already reported as biomarkers for resistance traits or for their involvement in grapevine fungal resistance, such as caffeic acid, catechin, epicatechin, leucocyanidin, quercetin-3-O-glucoside and derivatives, udihydroquercetin, dodecanoic acid and hexadecanoic and myo-inositol derivatives.

Ciubotaru and colleagues (Ciubotaru et al., 2023) studied alterations in primary and secondary metabolism, in response to *E. necator* inoculation, in mono-*locus* resistant genotypes, pyramided resistant genotypes and a susceptible cultivar. These authors identified several compounds that were up-accumulated in resistant genotypes and were not found in the susceptible one. Of the ten compounds that discriminated between resistant and susceptible genotypes, pallidol, oleic acid + *cis* vaccenic acid and astringin (Table 1), have been previously discussed as potential biomarkers of grapevine resistance to *P. viticola* (Ciubotaru et al., 2021). Overall, this study indicates that there might be a link between genotype and/or resistance *loci* and a cultivar’s behaviour to pathogen attack. Even though a similar metabolomic response was observed between the mono-*locus* and pyramided genotypes that share the same *loci*, there was no direct relationship between the number of resistance *loci* present and the production of the identified metabolite biomarkers.

#### 3.1.3 Grey mold disease

Grey mold disease is caused by the necrotrophic fungus *Botrytis cinerea*, that kills the host plant cells and colonizes the dead tissues, causing soft rotting of all aerial tissues and also rotting in post-harvest fruits, even in cold-stored products. *B. cinerea* is a globally widespread pathogen, lacking a specific host. Grey mold, also known as botrytis bunch rot in grapevine, can cause heavy losses in yield and quality in table and wine grapes (Armijo et al., 2016; AbuQamar et al., 2016).

Like downy and powdery mildew diseases, grey mold management relies on the application of chemical treatments at specific vine growth stages, raising the same concerns of development of pathogen resistance and public health safety risks associated with fungicide residues (De Miccolis Angelini et al., 2014). Most common *V. vinifera* cultivars show low or no resistance to grey mold. Several studies using metabolomics approaches have reported significant metabolic changes in grapevine leaves and berries caused by *B. cinerea* infection (see major changes in Table 1). Through a metabolic profiling using ^1^H NMR spectroscopy there were revealed clear differences between healthy berries taken from both healthy and botrytized bunches, and from botrytized berries (Hong et al., 2012). Pulp and skin analyses revealed that d-Gluconic acid was only detected in botrytized berries and accumulated more in the skin than in the pulp. Furthermore, skin samples from botrytized berries showed higher levels of alanine, glutamate, succinate, fructose, and glucose compared to skin samples retrieved from healthy bunches. In pulp, higher levels of valine, isoleucine, threonine, proline, glutamine, and glutamate were found in healthy berries from botrytized bunches compared to berries from healthy bunches. Also, the levels of succinate, arginine, and γ-aminobutyrate were higher in botrytized pulp compared to that of healthy bunches, and glycerol levels were increased in both skin and pulp of infected berries.

Agudelo-Romero and co-workers (Agudelo-Romero et al., 2015) described changes in the transcriptome and metabolome of grapes infected with *B. cinerea*, that suggest the activation of a defence response involving JA, ethylene, polyamines, and auxins. Grape primary metabolism, mainly carbohydrate and lipid metabolisms, appeared to be redirected toward the synthesis of precursors of defence-related metabolites. These authors were able to highlight several metabolites that, alone or combined, could be used as early infection biomarkers, such as gallic and azelaic acids, arabitol, ribitol, 4-amino butanoic acid, 1-O-methyl-glucopyranoside and several fatty acids (Table 1).

Given the effects of *B. cinerea* infection on grape quality, researchers have also been investigating the influence of this infection on the chemical composition of grape juices and wines. In a study using a GC–MS untargeted metabolomics approach, Schueuermann and co-workers (Schueuermann et al., 2019) compared the differences in the volatile profiles of juices from grapes infected with different fungal pathogens. The results showed that *B. cinerea* infected samples had higher concentrations of 1,5-dimethylnaphthalene and several unidentified sesquiterpenes and sesquiterpene fragments. These compounds allowed the discrimination of these samples from other fungal infections, showing their potential as volatile fungal markers for *B. cinerea*. It was also possible to distinguish between healthy and botrytized Champagne base wines by using ^1^H NMR-based metabolic profiling (Hong et al., 2011). The botrytized samples presented lower levels of glycerol, 2,3-butanediol, succinate, tyrosine, valine derivative, and phenylpropanoids and higher levels of oligosaccharides, indicating that the *B. cinerea* infection of grape berries affected the alcoholic fermentation processes, ultimately influencing wine quality.

#### 3.1.4 Grapevine trunk diseases

Grapevine trunk diseases (GTDs) are highly destructive diseases affecting vineyards worldwide, causing serious economic losses in the wine industry. Esca complex, Eutypa dieback, and Botryosphaeria dieback are the predominant GTDs. These diseases are generally characterized by a progressive decline of grapevines, caused by fungal pathogens (usually different species of a main pathogen or a complex of causal pathogens) that attack the perennial organs, leading to leaf and berry symptoms that result in a loss of productivity and eventual death of the plants (Reveglia et al., 2022; Bertsch et al., 2013).

GTDs diagnosis can be difficult due to the complexity of the symptomatology. After pathogen infection, it can take years before the appearance of the first symptoms, and the possibility of the presence of multiple GTDs in a single plant only adds to the difficulty of disease diagnosis (Reveglia et al., 2022). Being considered slow-progression diseases, older vineyards tend to show the most severe symptoms. Even though there are no grapevine taxa known to be resistant, the susceptibility to GTDs differs among cultivars (Reveglia et al., 2022; Bertsch et al., 2013).

The biggest challenge regarding GTDs is that there are no efficient curative methods for these diseases. The only effective treatment previously used was based on the application of sodium arsenite, which was banned in 2001 due to its toxicity to the environment and its carcinogenic effects in humans. Therefore, GTDs management currently relies on prophylactic measures, such as sanitation methods by applying fungicide treatments on pruning wounds, or remedial surgery or trunk renewal to remove the infected wood (Reveglia et al., 2022).

In recent years, many research teams have been studying GTDs through metabolomics approaches, comparing healthy and diseased samples and assessing grapevine-pathogen interactions. Lima and co-workers (Lima et al., 2017) studied the phenolic content of grapevine leaves affected with esca disease, comparing healthy leaves from asymptomatic cordons with apparently healthy and diseased leaves from affected cordons. Their results showed an increase in total phenolic production in response to the disease. Several compounds, such as trans-caffeoyltartaric acid, trans-coumaroyl-tartaric acid, quercetin-3-O-glucoside, quercetin-3-O-galactoside, kaempferol-3-glucoside and myricetin, exhibited a similar increase (Table 1). These changes in phenolic production were detected even in apparently healthy leaves from affected cordons, suggesting that this approach could be used to develop a method capable of detecting esca disease in plants not showing external symptoms (Lima et al., 2017).

Other studies in esca-affected grapevines have also highlighted an increase in the phenolic contents in wood lesions. Esca-associated fungi significantly increased the phenolics content in differently decayed wood from different trunk parts (Rusjan et al., 2017). It was further demonstrated that brown-red discoloured wood had higher concentrations of stilbene polyphenols than asymptomatic wood from esca-affected grapevines (Amalfitano et al., 2011). The most abundant stilbene polyphenols accumulated in symptomatic wood were ε-viniferin and resveratrol, suggesting that resveratrol and related compounds could be involved in the regulation of esca fungi-grapevine interaction.

In a study to gain insight on the interaction between grapevine and botryosphaeria dieback pathogens, Lemaitre-Guillier and colleagues (Lemaitre-Guillier et al., 2020) compared the metabolomic fingerprints of healthy and brown stripe affected wood of three cultivars. Their results showed that the disease had different impacts among the cultivars and significant differences were found between healthy and affected wood. This comparison revealed a higher accumulation of lipids in the asymptomatic areas close to the brown stripe, and a higher accumulation of secondary metabolites such as stilbenes in the symptomatic areas (Table 1).

Using an untargeted lipidomics approach to analyse grapevine leaves with varying degrees of esca-induced damage, Goufo and Cortez (Goufo and Cortez, 2020) showed that distinct metabolic pathways were stimulated at different stages of the disease development. The reported increase of some galactolipids and diacylglycerolipids in asymptomatic leaves and their progressive decrease with increasing leaf symptom severity, suggested that these lipid species may play role in the suppression of symptom expressions, by maintaining membrane integrity and normal protein function during the disease latency period, and stimulating the activation of grapevine defence mechanisms. The metabolite profiling of two ‘Chardonnay’ clones showed a clone-dependent metabolite fingerprint associated to esca disease expression, with opposing variations on the accumulation of specific metabolites in healthy and diseased samples of each clone (Moret et al., 2019).

With the goal of gaining a better understanding of the tolerance mechanisms of grapevine to GTDs, the wood metabolomic responses were compared between the susceptible *V. vinifera* subsp. *vinifera* and the more tolerant subsp. *sylvestris*, upon inoculation with *Neofusicoccum parvum*, one of the most aggressive fungus associated with botryosphaeria dieback (Labois et al., 2020). *N. parvum* inoculation triggered major changes in both primary and specialized metabolites in the wood of both subspecies. Overall, *V. vinifera* subsp. *sylvestris* showed a more rapid and intense alteration in primary metabolites and a higher induction of several resveratrol oligomers, which could be related to its increased tolerance to GTDs.

#### 3.1.5 Grapevine viral diseases

Viral diseases in grapevine are highly complex, mostly due to the occurrence of simultaneous infection by multiple viruses and the intricate nature of the compatible pathogen-host interactions, since virus resistance in grapevine is rare. These diseases, characterized by deformations in vegetative organs and foliar discolorations, can cause decreases in crop quantity and quality that result in significant economic losses. Under natural conditions, grapevine viruses are transmitted by diverse vectors, such as nematodes, mealybugs, or soft scale insects. Long-distance dissemination of viral diseases occurs mainly due to the use of infected propagation and planting material (Armijo et al., 2016; Fuchs, 2020). Current management of grapevine virus diseases relies on preventing and reducing the virus inoculum within the vineyard, using integrated strategies targeting both virus and its vectors. Replacing infected vines with clean planting material, including vector-tolerant rootstocks, if possible, is essential to prevent virus dissemination (Fuchs, 2020).

Recent advancements in diagnostic and sanitation methodologies have greatly improved the production process and quality of virus-free planting material, however there is still a need for new methods for swift on-site diagnosis of infected plants. In recent years, as an effort to develop early diagnostic methods, metabolomic approaches have been employed to uncover suitable parameters that could work as indicators of virus presence in asymptomatic plants (Fuchs, 2020; Mandrile et al., 2022; Montero et al., 2016). Through metabolic profiling of grapevine leaf samples from healthy and asymptomatic plants infected with Grapevine leafroll-associated virus 3 (GLRaV-3), Montero et al. (2016) identified changes in the metabolism triggered by the virus that could be responsible for alterations in fluorescence parameters. These authors suggest that fluorescence imaging could be used as an on-site tool for discrimination of GLRaV-3 infected plants at very early stage of infection, by identifying specific disease signatures. Raman spectroscopy was also able to discriminate healthy plants from those infected by Grapevine fan leaf virus (GFLV) and Grapevine rupestris stem pitting-associated (GRSPaV) (Mandrile et al., 2022). These authors identified changes in the metabolism triggered by GFLV and GRSPaV with an accuracy up to 100% and 80%, respectively, even in the absence of visible symptoms. The analysis of the obtained spectra effectively detected changes in carotenoid levels in infected grapevine leaves, which are more profoundly altered by GFLV infection. This study shows the potential of Raman spectroscopy as a tool for early virus detection in vineyards and nurseries, and future research aims to use high-throughput portable Raman spectrometers for direct assessment plants in the field.

### 3.2 Abiotic stresses

Climate changes like variations in rainfall, heat waves and global CO_2_ levels are responsible for several types of abiotic stresses, causing a negative impact in global agriculture and food production. Metabolomics, due to its proximity to the phenotype and considered highly environmentally sensitive, is a key tool for assessing biochemical changes in plants affected by abiotic stress (Shulaev et al., 2008), allowing to elucidate the mechanisms for tolerance to abiotic stress in plants.

#### 3.2.1 Drought

Drought is one of the main environmental constraints affecting plant survival, limiting the productivity of agricultural crops worldwide. Furthermore, due to climate change, crop growth models predict that this issue will be more severe in future, leading to frequent periods of drought as well as threats to both natural and agricultural ecosystems (Gornall et al., 2010).

Plants regulate the balance of their physiology, morphology and metabolism to survive water stress. The responses of plants under water deficit stress occur from the leaf to the whole plant level and require the establishment of a new state of cellular homeostasis, leading to a change in the metabolic profile (Anjum et al., 2011). Thus, it is important to investigate the changes in the photosynthetic parameters, proline content, reactive oxygen species (ROS) levels, and antioxidant enzyme activities in response to water deficit stress.

The effect of drought stress at plant and fruit level has been investigated on grapevine metabolome (major changes are shown in Table 1). Because grapevines are frequently cultivated in arid and semi-arid areas (Zarrouk et al., 2015), it is critical to study their responses to water stress. Indeed, this type of stress influences the primary and secondary metabolism of plants, having impacts on the content of carbohydrates, and secondary metabolites as, e.g., polyphenols and volatile organic compounds (VOCs). Regarding primary metabolites, an increase in myo-inositol, sucrose and alanine was observed under water-deficit stress in the pulp of grape berries, whereas glutamate and tartrate were more abundant in the pulp of berries from well-watered vines (Grimplet et al., 2009). Grimplet and co-authors (Grimplet et al., 2009) suggested that the reduction of glutamate and increase in alanine in pulp of water-stressed plants indicates that glutamate decarboxylase is involved in metabolic adjustments under water stress. Furthermore, the large increase in myo-inositol and sucrose under water-deficit seems to indicate their roles as osmoprotectants and precursors for the formation of sugars to enhance water-deficit stress tolerance (Grimplet et al., 2009) (Table 1). On the contrary, in drought stressed grapevine leaves, sucrose was not significantly influenced, maybe reflecting a different adaptation in leaves comparing to berries (Griesser et al., 2015). Additionally, a significant increase of proline in grapevine leaves (Doupis et al., 2011; Hochberg et al., 2013; Ju et al., 2018) and berries (Deluc et al., 2009) under drought conditions has been observed. Proline participates in protection against the formation of excessive ROS and, consequently, improving the resistance to drought stress (Szabados and Savouré, 2010).

Under drought stress, the production of ROS molecules such as H_2_O_2_ acts as a signal, enhancing the resistance of grapevines to drought (Vidigal et al., 2013). As a result of increased ROS, also the activity of some antioxidant enzymes’ changes. Previous studies reported an increase in superoxide dismutase and catalase (CAT) activity in grapevine leaves, which indicates that grapevines increase the activities of these antioxidant enzymes to scavenge ROS (Ju et al., 2018).

In the case of secondary metabolites, Green leaf volatiles (GLVs), which play an important role in plant defense against herbivory, showed an increase in their concentration in drought-stressed grapevine leaves (Ju et al., 2018). Also phenolic compounds, essential players regarding grape and wine quality, have an important role in plant defense against abiotic stresses. Polyphenols are protective molecules against oxidative damage by scavenging ROS produced during stress (Sharma et al., 2019). Several studies have shown an influence of water stress on the different classes of polyphenols in the skins of mature grape berries, depending on cultivar, with different compounds affecting either positively or negatively grape and wine quality (Sharma et al., 2019; Ollé et al., 2011).

#### 3.2.2 Salinity

Salinity is one major abiotic stress, affecting 7% of land area and 33% of irrigated lands worldwide, with a tendency to increase each year. Salinity stress has been defined as the accumulation of salts in the rhizosphere, predominantly sodium (Na^+^) and chloride (Cl^*−*^) ions (reviewed by Chele et al. (2021)). In general, when the electrical conductivity of the saturation extract in the root zone is higher than 40 mM at 25°C, with 15% of unbound Na^+^ ions, soil is considered saline (Shrivastava and Kumar, 2015). Agricultural malpractices, such as overuse of chemical fertilizers, bad irrigation practices and industrial pollution, along with climate change that contribute to low precipitation, lead to a greater salt concentration in the soil upon evaporation. When water evaporates, the salt ion residue is left around the roots, inhibiting the uptake of water and nutrients, impairing plant development (Chele et al., 2021). Under saline conditions, plants activate different physiological and biochemical mechanisms, such as changes in morphology, anatomy, photosynthesis, hormonal profile, toxic ion distribution and biochemical adaptation. These diverse strategies depend on several factors, such as the species, plant age and size, the intensity as well as the duration of the stress exposure (Acosta-Motos et al., 2017).

Most grapevines grow in arid or semi-arid areas, where soil salinization, associated with low rainfall and high evaporation, are a concern. Excessive salinity can cause multiple negative effects on viticulture and winemaking (Walker et al., 2002). Grapevine roots frequently accumulate a huge amount of salt, which affects the normal growth and development of the plants, resulting in yield loss (Walker et al., 2002). In a recent study, Lu and co-workers (Lu et al., 2022) analyzed the metabolome of grape leaves and showed that 431 differentially accumulated metabolites (DAMs) were identified in salt stress. The authors highlighted that salt stress in grape plants negatively affected the Calvin cycle in carbon fixation, increasing the abundance of metabolites related with this pathway. On the contrary, Rubisco activity and sugar metabolites decreased under this stress condition (Table 1). Sugar metabolites are the main products of photosynthesis, playing a key role in stress perception and signaling, and form a regulatory center for gene expression mediated by adverse environments, thus ensuring osmoregulation responses, scavenging ROS, and maintaining cellular energy status through carbon partitioning (Saddhe et al., 2021).

Chlorophyll levels decrease under high NaCl concentration, suggesting an inhibition in the electron transport of photosystem II, probably associated with membrane lipid peroxidation due to ROS (reviewed by Zhou-Tsang et al., 2021). Also, metabolites related with chlorophyll synthesis, as L-Glutamic acid and 5-Aminolevulinate, decrease under salt stress (Lu et al., 2022). The abundance of organic acids increased in response to Na^+^ homeostatic imbalance, perhaps with the function of neutralizing excess cations to maintain charge balance (Lu et al., 2022) (Table 1).

Nevertheless, the antioxidant capabilities of grapevine are well known. Several studies demonstrated the antioxidant response against salt stress in *V. vinifera* and the results showed an increase in CAT and ascorbate peroxidase activity and total phenolic content in leaves and roots after saline irrigation (reviewed by Zhou-Tsang et al., 2021).

#### 3.2.3 Ultra violet and infrared radiation

Climate change is altering weather patterns, leading to changes in light and temperatures regimes while also causing an increase in drought prevalence (Blunden and Boyer, 2021). For terrestrial plants, light is not only an important source of energy, but it is also an important medium of transfer information from environment to plants. Of the total solar radiation that reaches earth’s surface, approximately 92% is composed of infrared radiation and visible light, while ultraviolet radiation accounts only 8% (Taiz et al., 2023).

Plants present sophisticated mechanisms to detect and interpret information from the surrounding environment, including variations in the electromagnetic spectra (Taiz et al., 2023). By detecting the variation in light environment, plants can optimize their growth and development, either through an effect on photosynthesis or by influencing photomorphogenesis. In the Mediterranean region there have been several significant extreme warm episodes in the last decade with high irradiance (about 3,000 sunshine hours per year) and high temperature (Blunden and Boyer, 2021).

Different types of photoreceptors have been identified in various plant species that play a fundamental role in the perception of light environment changes: the cryptochromes are sensible to blue light (Banerjee and Batschauer, 2005), the red/far-red light-sensing phytochromes (Sharrock, 2008) and the UVB photoreceptor UVR8 (UV RESISTANCE LOCUS 8) (Zhang et al., 2022). Although UV radiation is a damage-inducing source of stress in plants, it can trigger various metabolic pathways that can positively influence crop productivity (Mansour et al., 2022).

Changes in metabolite synthesis induced by sunlight exposure are not easily predicted among plant species and there are some contradicting results. In *V. vinifera* the increase of flavonoid content in grape berries has been widely reported in response to UV-B exposure (Carbonell-Bejerano et al., 2014; Liu et al., 2015; Loyola et al., 2016; Rouxinol et al., 2023) (Table 1). However, the effect of increased radiation is not always accompanied by higher contents of polyphenols in berry skins. For instance, as a consequence of sunlight exposure after veraison, it was observed a reduction in anthocyanin, flavonol and sugar content in grape berries compared with non-exposed grapes (Matus et al., 2009) (Table 1). Furthermore, the conjugation of different abiotic stresses, including drought, can lead to a lower capacity of grapevines to cope with high UV radiation, resulting in lower photosynthetic rate, physiological damages and lower production (Rouxinol et al., 2023).

The composition of epicuticular layer has been associated with plant response to water stress due to its hydrophobic properties. In grape berries this natural barrier is composed by lipophilic substances such as terpenes, alkanes, esters aldehydes and fatty acids (Yang et al., 2021). In high UVB irradiance, vines react by producing diterpenes with antioxidant properties and abscisic acid (ABA) depending on the UVB flux rate. Instead, in low UVB concentration regime there was an intensified *de novo*-synthesis of sesquiterpene E-nerolidol as well as the monoterpenes a-pinene, terpinolene and careen, as determined by chromatography coupled to electron impact mass spectrometry (GC-EIMS) (Gil et al., 2012) (Table 1). Also, the outermost layer of leaves is composed of hydrophobic substances: fatty acids and substances derived from fatty acids (Kerstiens, 1996). Plants of *V. vinifera* responded to water stress conditions by increasing the very long chain fatty acid content in leaf layer epicuticular lipids during the growing season (Spangenberg et al., 2020). Also, differences in the accumulation of lipids on the leaf cuticle surface were registered among *V. vinifera* cultivars, suggesting that a higher rate of wax accumulation in the leaves will be an advantage in stress conditions (Spangenberg et al., 2020).

Carotenoid accumulation plays a crucial role in the photoprotection of grape berries. These compounds are efficient antioxidants capable of scavenging singlet oxygen (^1^O_2_^*^) and peroxyl radicals. Additionally, they quench triplet chlorophyll (^3^Chl^*^) generated during photooxidation processes and have the ability to screen light in the blue-green and UV parts of the spectrum (Gambetta et al., 2021). In the green berry stage, violaxanthin, anteraxanthin, zeaxanthin and neoxanthin increase rapidly in response to high light exposures (Kiselev et al., 2019) (Table 1). In the presence of UV-C radiation, stilbenes such as resveratrol are synthetised to protect vine leaves against UV radiation and this synthesis was more intense when combined with p-coumaric acid application, indicating that this phenolic precursor intensified the stilbene biosynthesis (Kiselev et al., 2019).

### 3.3 Induced morphogenic processes

Induced plant morphogenesis is an agronomical trait that involves a cell reprogramming process led by changes in gene expression patterns, with expression at the biochemical and physiological levels, ending with *de novo* differentiation of a new organ. *De novo* morphogenesis includes processes with high agronomical impact, such as the efficient development of adventitious roots (adventitious rooting) and the differentiation of somatic embryos, both described as a morphogenic response to abiotic stress factors (Potters et al., 2007).

#### 3.3.1 Adventitious rooting

The process of adventitious rooting (AR) involves the differentiation of roots from differentiated cells belonging to a different organ, usually a stem cutting (herbaceous, softwood, semi-hardwood or hardwood), and is considered a critical point in the clonal propagation of diverse agronomically important plant species. In grapevine, the ability to develop adventitious roots is a vital determinant of the successful production of high-quality grapevine planting material (Anhalt et al., 2011; Riaz et al., 2002). Research focused on AR in grapevine has mainly concentrated on applied and physiological aspects (Kaur et al., 2002), but fundamental research on the molecular mechanisms underlying AR process in recalcitrant and non-recalcitrant cultivars is lacking on grapevine.

The phytohormones, such as auxin and ethylene, are two main players in the AR process. A auxin plays a pivotal role in the induction of the process while ethylene has been implicated in the biosynthesis and response to exogenous auxin application (Ivanchenko et al., 2008). In ‘Mangio’ (*V. riparia* Mchx x *V. rupestris* Scheele) a peak of ethylene production was detected in microcuttings at an early stage of the AR process, and its potential involvement in the initiation of network reactions leading to root formation was hypothesized (Moncousin et al., 1989). A second ethylene peak detected at a later rooting stage during the induction phase was associated with the beginning of the indole-3-acetic acid (IAA) increasing (Moncousin et al., 1989). IAA is the most common auxin in plants, and its involvement in AR has been highly explored across plant species, mostly by using IBA which is *in cell* converted to IAA (Cardoso et al., 2022; Ribeiro et al., 2022; Velada et al., 2020). Using hardwood cuttings of different rootstocks (5BB–*V. berlandire* x *V. riparia*, 41B–Chasselas x *V. berlandiere*, and 420A–*V. berlandieri* x *V. riparia*), Kelen and Ozkan (Kelen and Ozkan, 2003) reported high IAA and low ABA levels associated with a high rooting rate, and low IAA and high ABA levels with a low rooting rate.

Lipid metabolism was also implicated in AR in grapevine. Kraiem and colleagues (Kraiem et al., 2010) reported a negative correlation between the root number and linoleic and linolenic acids when using apical cuttings of *V. vinifera* cv. ‘Carignan’ but in contrast, palmitic, palmitoleic, stearic and oleic acids correlated positively with the root number.

#### 3.3.2 Somatic embryogenesis

Somatic Embryogenesis (SE) is a morphogenic process where embryogenic structures similar to zygotic embryos are obtained from somatic cells, and are able to develop into complete individuals. In woody species the application of SE provides many benefits, especially if applied to propagation of species with a recalcitrant behavior to the conventional propagation methods (Awada et al., 2023; Martínez et al., 2019). Nevertheless, the establishment of *in vitro* protocols for plant regeneration by SE requires previous optimizations considering multiple exogenous and endogenous factors (Cardoso et al., 2010; Cardoso et al., 2019). Considering the roles played by metabolites in plants, secondary metabolites can reflect the physiological phenomena in the explants used, regulate gene transcription and protein synthesis (Guo et al., 2019), and, at the same time, may be responsible for the differences on the ability to positively respond to the embryogenic stimulus.

Faure and Aarrouf (Faure and Aarrouf, 1994), studying the metabolism of reserve products during somatic embryos’ development of *V. vinifera* cv. ‘Grenache noir,’ reported the accumulation of high levels of lipid bodies and low levels of starch at the torpedo stage, but at the following developmental stages, starch and lipids accumulate but are not properly used, partially due to the absence of isocitrate lyase activity necessary to the glyoxylate cycle. Later, Faure et al. (1998) also studying the involvement of ABA and IAA on embryogenesis, further conversion and germination of embryos into SE, reported that the low efficiency seen on embryos conversion and germination was linked to the inexistence of a peak of ABA accumulation during embryogenesis leading to the inability to switch from mid-to late-embryogenesis, and concluded that the accumulation of ABA and IAA must occur in sufficient concentrations to allow normal plantlet development. More recently, Acanda and co-workers (Acanda et al., 2020) showed that in *V. vinifera* cv. ‘Mencía’ somatic embryos, the levels of endogenous ABA and ABA-glucosyl ester showed two peaks during embryos’ culture, demonstrating the involvement of ABA metabolism in the control of maturation of grapevine somatic embryos.

Pectin, a polysaccharide that represents the major component of the primary plant cell wall and intercellular layer, has an essential role for normal cell growth and the establishment or maintenance of cell differentiation (Barnes and Anderson, 2018). Yu and colleagues (Yu et al., 2023), working with pro-embryonic masses of cv. ‘Thompson Seedless’ maintained under *in vitro* culture, demonstrated that the decrease in the cell walls of esterified and de-esterified pectin contents was probability related to the loss of embryogenesis ability during long-term subculture of pro-embryonic masses. Furthermore, it was also shown that polyamine metabolism, particularly putrescine metabolism, is involved in the correct maturation of grapevine somatic embryos (Domínguez et al., 2023).

## 4 Grapevine holobiont characterization

In plants, the assumption of a direct link between genotype and phenotype is no longer valid. In fact, the interrelation between plants and their associated endo- and extracellular symbiotic microbiome cannot be dissociated from the phenotype (Zilber-Rosenberg and Rosenberg, 2008). These microorganisms, which include a wide array of fungi, bacteria, actinomycetes, algae, and protozoa, may influence the plant phenotype by buffering environmental changes. A more holistic perception of plants as entities in permanent interaction with their microbiome is therefore gaining strength. This holobiont entity, an additional organismal level, has to develop mechanisms that can accommodate phenotypic plasticity in regard to environmental conditions, both from the host as from the symbiont.

The plant microbiome is involved in major functions such as enhance of plant nutrition and plant resistance to biotic and abiotic stresses (Trivedi et al., 2020; Bergelson et al., 2019). Multiple functions are performed by these diverse microbial communities, such as nitrogen fixation and solubilisation (Kour et al., 2021; Mahmud et al., 2020), production of phytohormones such auxins, cytokinins (CKs), ABA, gibberellins (GAs), and SA (Egamberdieva et al., 2017), protection against pathogens and recruitment of other beneficial microorganisms (Kwak et al., 2018; Gao et al., 2021).

Recent research has indicated that metabolites in plants can shape the plant microbiome (Jacoby et al., 2021; Huang et al., 2019) and plant microbiota can also influence the phytometabolome (Etalo et al., 2018). Understanding the dynamics of the holobiont metabolome can provide a better molecular insight into the outcome of the plant symbioses.

### 4.1 Microbiome-driven changes in plant metabolome

The type and abundance of compounds naturally excreted by plants are dependent on the plant species and genotype, the developmental stage, plant management and the existence or not of a stress factor (Trivedi et al., 2020). Plant root exudates comprise both primary and secondary metabolites, and can mediate the type of associated microbiota (Hu et al., 2018). On the other hand, biotic interactions can mediate the type of compounds excreted by plants.

Although the plant microbiota includes a wide diversity of microorganisms, the majority of research has focused on bacterial and fungal communities (Trivedi et al., 2020). Plant growth promoting bacteria (PGPB) live mostly inside the rhizosphere and/or roots of plants, but can also be isolated from the surface of other plant tissues (epiphytic) (Andreolli et al., 2021; Souza et al., 2015; Arun et al., 2020). The mechanisms by which PGPB stimulate plant growth involve direct mechanisms such as nitrogen fixation, phosphate solubilisation and production of phytohormones, and indirect mechanisms like production of siderophores and antibiotics, modulation of ACC deaminase expression, and competition with pathogenic bacteria (Kour et al., 2021; Egamberdieva et al., 2017; Krewulak and Vogel, 2008; Arshad et al., 2008; Ajijah et al., 2023; Zhou et al., 2021).

Similarly, fungi play an important role in natural and agricultural ecosystems. Aside from pathogenic fungi, the benefits of plant-fungal interactions have been amply demonstrated both for endophytic as epiphytic fungi, with positive effects on plant nutrition, stress tolerance and disease resistance (Genre et al., 2020; Rodriguez et al., 2009).

#### 4.1.1 Implications of microbiome-induced metabolome for the biocontrol of grapevine diseases

The grapevine microbiota is diverse and rather different amongst above- and belowground organs. It was shown that the composition of the aboveground compartments is partially shaped by the vineyard soil (Zarraonaindia et al., 2015), along with the atmospheric microbiota (Abdelfattah et al., 2019). Several studies showed that the root microbiota is more diverse than that of the aerial parts and enriched in *Proteobacteria*, *Acidobacteria*, *Actinobacteria*, *Chloroflexi*, *Bacteroidetes* and *Verrucomicrobia* spp (Zarraonaindia et al., 2015; Marasco et al., 2018). *Proteobacteria* are highly dominant in flowers, grapes and leaves (Zarraonaindia et al., 2015). Interestingly, the core mycobiota of grapevine roots was shown to change over the growing season (Liu and Howell, 2021), as, for example, for *Aureobasidium*, *Cryptococcus* and *Cladosporium* species.

In grapevine, many microorganisms of great concern for plants’ health are associated with aerial organs (Mondello et al., 2018) and were shown to alter grapevine metabolic profile. For instance, fungal infections causing trunk diseases such as Esca, Botryosphaeria dieback, Eutypa dieback and Phomopsis dieback cause changes in phenolic compounds during the progress of the infection (Galarneau et al., 2021). Research on the use of beneficial plant microorganisms for the control of trunk diseases highlighted the potential use of Actinobacteria, particularly of the genus *Streptomyces*, which produces a wide range of antibiotics and volatile organic compounds (Vurukonda et al., 2018; Cobos et al., 2022). Vatsa-Portugal and co-workers (Vatsa-Portugal et al., 2017) showed that a strain of *S. anulatus* induced the upregulation of genes related to secondary metabolism and enhanced the production of stilbenic phytoalexins in grapevine cells challenged with *B. cinerea*, the causing agent of grey mold. Interestingly, a priming effect for enhanced plant defense reaction upon *B. cinerea* challenge has been observed for other non-pathogenic rhizobacteria such as *Pseudomonas fluorescens*, with an induction of genes related with secondary metabolism such as *phenylalanine ammonia-lyase* (*PAL*), *stilbene synthase* (*STS*), *glutathione* S-*transferase* (*GST*), *lipoxygenase 9* (*LOX9*) and *1-aminocyclopropane-1-carboxylic acid-synthase* (*ACCsyn*) (Gruau et al., 2015). PAL catalyzes the initial reaction of phenylpropanoid metabolism and cinnamate synthesis, the SA precursor, which causes systemic resistance in plants (Huang et al., 2010; Kim and Hwang, 2014). STSs catalyse the biosynthesis of stilbenes, which are a small family of phenylpropanoids produced in some plant species, including grapevine. In fact, stilbenic compounds, including resveratrol and its dehydrodimer ε-viniferin, can be triggered by various non-pathogenic rhizobacteria and are associated with systemic induced resistance of plants to pathogens (Vatsa-Portugal et al., 2017; Gruau et al., 2015; Aziz et al., 2016).

As for other plant species, it is well established that the association between grapevine and arbuscular mycorrhizal fungi (AMF) provides benefits for the plant, particularly enhancing the uptake of nutrients and protecting against biotic and abiotic stresses (Trouvelot et al., 2015). Also, plant–AMF symbiosis is known to reprogram the primary and secondary metabolism in plants (Kaur and Suseela, 2020; Weng et al., 2022). Nogales and colleagues (Nogales et al., 2009) showed that grapevines inoculated with the AMF *Rhizoglomus irregulare* had a better performance when infected with the root-rot fungus *Armillaria mellea* than non-inoculated plants, and it was suggested that early changes in polyamines levels could relate to an enhanced protection of AMF-inoculated plants.

#### 4.1.2 Implications of microbiome-induced metabolome for abiotic stress resistance

The interactions between the plant, the microbiome and the surrounding environment are complex, but an increasing number of studies are unravelling how beneficial microorganisms can affect plant phenotype regarding stress factors. Plant genotype also seems to influence the associated microbiota upon stress. For example, the enhanced tolerance of some grapevines cultivars to salinity might also be due to specific rhizosphere microbiome changes after exposure to salt stress (Wang et al., 2023).

Grapevines are often exposed to a variety of abiotic stresses, where important changes in primary metabolism occur (Hochberg et al., 2013). In rice it was shown that the disaccharide trehalose can provide the plants stress endurance, by forming a gel during cellular dehydration, thus efficiently stabilizing dehydrated enzymes, proteins and lipid membranes (Garg et al., 2002). Inoculation with PGPB can reduce the damaging effects of salt or drought stress by increasing the levels of trehalose in plants (Forni et al., 2017), and interestingly in grapevine, the beneficial effects of *Paraburkholderia phytofirmans* strain PsJN against cold stress seemed also to be related to an increase on trehalose metabolism (Fernandez et al., 2012).

AMF also contribute for drought stress alleviation in grapevine as indicated by lower levels of proline in AMF-colonized plants (Pavithra and Yapa, 2018). A recent study further showed that grapevine colonization with *R. irregulare* triggered major reprogramming of primary metabolism in the roots and to a lesser extent in leaves (Goddard et al., 2021). Particularly, the sugar and fatty acid metabolisms were strongly altered in roots, as, for example, several fatty acids involved in JA biosynthesis (linoleic and linolenic acids) were greatly enhanced in colonized roots (Goddard et al., 2021). Furthermore, leaves of colonized plants had higher levels of JA and SA than non-colonized plants and a higher expression of *VvAOS5*, than can be involved in the synthesis of the JA precursor 2-oxophytodienoic acid (12-OPDA) (Goddard et al., 2021). Jasmonate molecules can coordinate many cellular activities, including plant growth and development and response to several stresses (Sohn et al., 2022). Campos et al. (2023) also showed that grapevine AMF colonization under heat stress altered the expression of several microRNAs with possible implications on diverse metabolic pathways.

Melatonin is a strong antioxidant that protect plants against ROS and also promotes plant growth (Hernández-Ruiz and Arnao, 2016). There is a substantial crosstalk between melatonin and plant hormones such as auxin, CKs, GAs, ABA, ethylene, JA and SA (Arnao and Hernández-Ruiz, 2018). Jiao and co-workers (Jiao et al., 2016) showed that endogenous melatonin accumulation occurred in grapevines inoculated with *Bacillus amyloliquefaciens* under drought or salt stress, with a concomitant decrease in the production of malondialdehyde and ROS (H_2_O_2_ and O_2_^−^) in roots.

Terpenoids are the largest group of plant secondary metabolites and known to be involved in plant stress responses (Boncan et al., 2020). Isolates of *B. licheniformis* Rt4M10 and *P. fluorescens* Rt6M10 were shown to retard water losses and induce the accumulation of ABA and the activation of metabolic pathways for defense-related terpenes such as α-pinene, terpinolene, 4-carene, limonene, eucalyptol, lilac aldehyde A, α-bergamotene, α-farnesene, nerolidol, and farnesol (Salomon et al., 2014).

## 5 Future perspectives

Despite advancements in grapevine plant metabolomics focused on biotic and abiotic stress research, additional studies are required to comprehend the mechanisms underlying plant response and acclimatization to further allow selection of genotypes characterized by its high plasticity upon environmental constrains. This understanding is highly important for plant breeding, allowing the development and propagation of genotypes multistress tolerant/resistant ensuring food security in the panorama of climatic changes. Moreover, the comprehension of grapevine microbiome cannot be dissociated from the plant phenotypic response, as plant metabolites are greatly modulated by its microbial communities.

The utilization of advanced analytical techniques, such as MS and NMR, has propelled metabolome research. The concurrent use of multiple techniques or detectors in online or parallel analysis shows promising, as it enables comprehensive metabolite coverage and enhances quantification limits. These techniques facilitate monitoring changes in concentrations of endogenous metabolites, providing insights into plants’ responses to environmental conditions.
